# Kernelized Heterogeneity-Aware Cross-View Face Recognition

**DOI:** 10.3389/frai.2021.670538

**Published:** 2021-07-20

**Authors:** Tejas I. Dhamecha, Soumyadeep Ghosh, Mayank Vatsa, Richa Singh

**Affiliations:** ^1^ IIIT Delhi, New Delhi, India; ^2^ IIT Jodhpur, Jodhpur, India

**Keywords:** face recognition (FR), discriminant analysis (DA), heterogeneity, cross-spectral, cross-resolution

## Abstract

Cross-view or heterogeneous face matching involves comparing two different views of the face modality such as two different spectrums or resolutions. In this research, we present two heterogeneity-aware subspace techniques, heterogeneous discriminant analysis (HDA) and its kernel version (KHDA) that encode heterogeneity in the objective function and yield a suitable projection space for improved performance. They can be applied on any feature to make it heterogeneity invariant. We next propose a face recognition framework that uses existing facial features along with HDA/KHDA for matching. The effectiveness of HDA and KHDA is demonstrated using both handcrafted and learned representations on three challenging heterogeneous cross-view face recognition scenarios: (i) visible to near-infrared matching, (ii) cross-resolution matching, and (iii) digital photo to composite sketch matching. It is observed that, consistently in all the case studies, HDA and KHDA help to reduce the heterogeneity variance, clearly evidenced in the improved results. Comparison with recent heterogeneous matching algorithms shows that HDA- and KHDA-based matching yields state-of-the-art or comparable results on all three case studies. The proposed algorithms yield the best rank-1 accuracy of 99.4% on the CASIA NIR-VIS 2.0 database, up to 100% on the CMU Multi-PIE for different resolutions, and 95.2% rank-10 accuracies on the e-PRIP database for digital to composite sketch matching.

## Introduction

**FIGURE 1:**
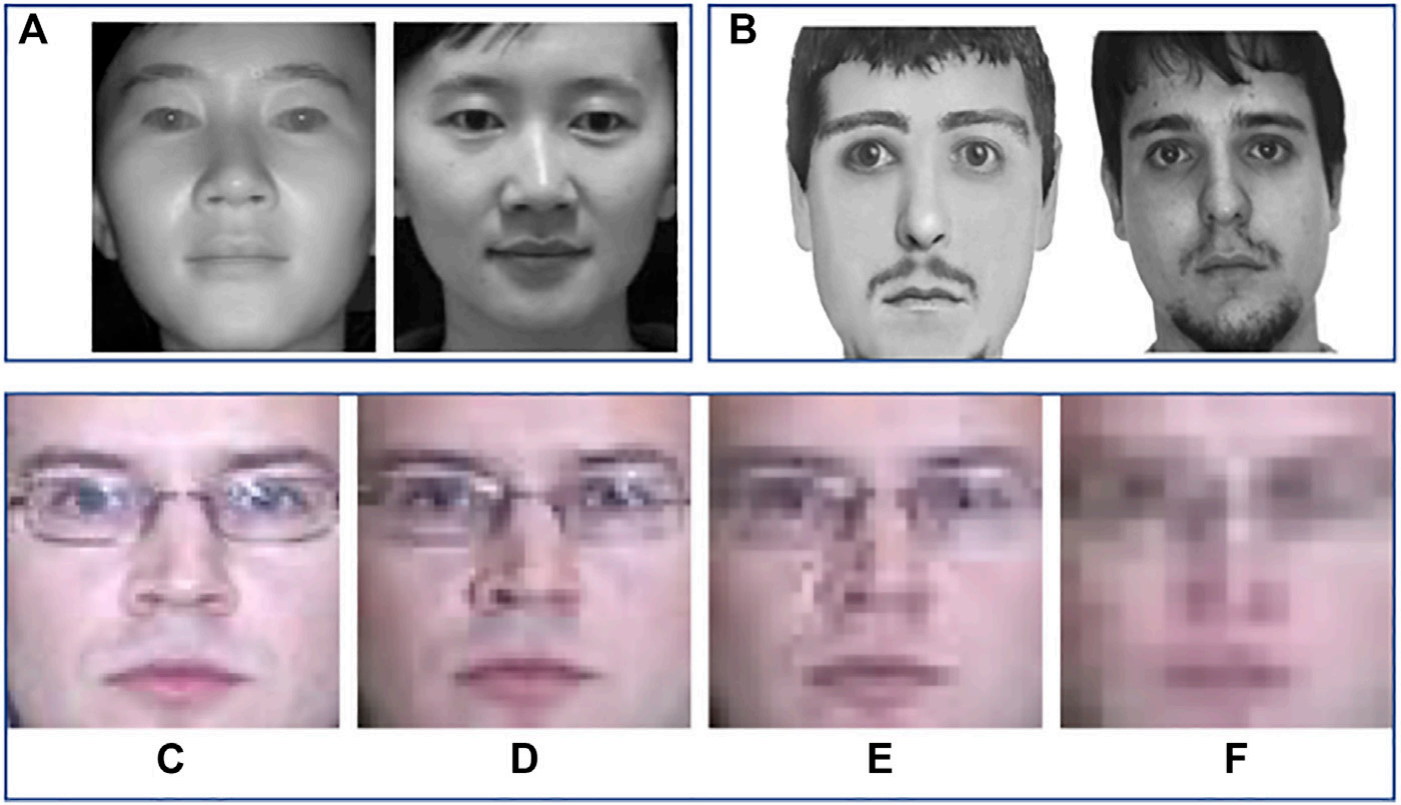
Examples of heterogeneous face recognition scenarios. Top row **(A)** shows heterogeneity due to difference in visible and near-infrared spectrum; **(B)** shows photo and composite sketches of a person. **(C)**–**(F)** illustrates heterogeneity due to resolution variation of 72x72, 48x48, 32x32, and 16x16, respectively. (The images of different resolution are stretched to common sizes.)

With increasing focus on security and surveillance, face biometrics has found several new applications and challenges in real-world scenarios. In terms of the current practices by law enforcement agencies, the legacy mugshot databases are captured with good quality face cameras operating in the visible spectrum (VIS) with inter-eye distance of at least 90 pixels (Wilson et al., 2007). However, for security and law enforcement applications, it is difficult to meet these standard requirements. For instance, in surveillance environment, when the illumination is not sufficient, majority of the surveillance cameras capture videos in the near-infrared spectrum (NIR). Even in daytime environment, an image captured at a distance may have only 16 × 16 facial region for processing. For these applications, the corresponding gallery or database image is generally a good quality mugshot image captured in controlled environments. This leads to the challenge of heterogeneity in gallery and probe images. Figure 1 shows samples of these heterogeneous face matching cases. This figure also showcases another interesting application of matching composite sketch images with digital face images. In this problem, composite sketches are generated using a software tool based on eyewitness description, and this synthetic sketch image is then matched against a database of mugshot face images. Since the information content in sketches and photos is different, matching them can be viewed as heterogeneous matching problem.

The challenge of heterogeneous face recognition is posed by the fact that the view^1^ of the query face image is not the same as that of the gallery image. In a broader sense, two face images are said to have different views if the facial information in the images is represented differently. For example, visible and near-infrared images are two views. The difference in views may arise due to several factors such as difference in sensors, their operating spectrum range, and difference in the process of sample generation. Most of the traditional face recognition research has focused on homogeneous matching (Bhatt et al., 2015), that is, when both gallery and probe images have the same views. In recent past, researchers have addressed the challenges of heterogeneous face recognition (Tang and Wang, 2003; Yi et al., 2007; Lei and Li, 2009; Lei et al., 2012a; Klare and Jain, 2013; Jin et al., 2015). Compared to homogeneous face recognition, matching face images with different views is a challenging problem as heterogeneity leads to increase in the intra-class variability.

### Literature Review

The literature pertaining to heterogeneous face recognition can be grouped into two broad categories: 1) heterogeneity invariant features and 2) heterogeneity-aware classifiers. Heterogeneity invariant feature–based approaches focus on extracting features which are invariant across different views. The prominent research includes use of handcrafted features such as variants of histogram of oriented gradients (HOG), Gabor, Weber, local binary patterns (LBP) (Liao et al., 2009; Goswami et al., 2011; Kalka et al., 2011; Chen and Ross, 2013; Dhamecha et al., 2014), and various learning-based features (Yi et al., 2015; Liu et al., 2016; Reale et al., 2016; He et al., 2017; Hu et al., 2018; Cho et al., 2020). Heterogeneity-aware classifier–based approaches focus on learning a model using samples from both the views. In this research, we primarily focus on designing a heterogeneity-aware classifier.

One set of work focuses on addressing the heterogeneity in projection space or by statistically learning the features suitable for heterogeneous matching. On these lines, one of the earliest research related to visible to near-infrared matching, proposed by Yi et al. (2007), utilizes canonical correlation analysis (CCA) which finds the projections in an unsupervised manner. It computes two projection directions, one for each view such that the correlation between them is maximized in the projection space. Closely related to CCA, Sharma et al. (2012) proposed generalized multi-view analysis (GMA) by adding a constraint that the multi-view samples of each class are as much closer as possible. Similar multi-view extension to discriminant analysis is also explored (Kan et al., 2016). Further, dictionary learning is also utilized for heterogeneous matching (Juefei-Xu et al., 2015; Wu et al., 2016). Efforts to extract heterogeneity-specific features have resulted in common discriminant feature extractor (CDFE) (Lin and Tang, 2006), coupled spectral regression (CSR) (Lei and Li, 2009) and its extensions (Lei et al., 2012a, b), common feature discriminant analysis (CFDA) (Li et al., 2014), coupled discriminative feature learning (CDFL) (Jin et al., 2015), and coupled compact binary face descriptors (C-CBFD) (Lu et al., 2015). Similarly, mutual component analysis (MCA) Li et al. (2016) utilizes iterative EM approach along with a modeling of face generation process to capture view-invariant characteristics.

Although statistical in spirit, a body of work approaches the heterogeneity challenge as a manifold modeling problem. These works explore manifold learning–based approaches to learn heterogeneity-aware classifier. Li et al. (2010) proposed locality preserving projections (LPP)–based approach that preserves local neighborhood in the projection space. Biswas et al. (2013, 2012) proposed a multidimensional scaling (MDS)–based approach for matching low-resolution face images. The algorithm learns an MDS transformation which maps pairwise distances in kernel space of one view to corresponding pairwise distances of the other view. Klare and Jain (2013) proposed a prototyping-based approach. It explores the intuition that across different views, the relative coordinates of samples should remain similar. Therefore, the vector of similarities between the query sample and prototype samples in the corresponding view may be used as the feature.

Other research directions, such as maximum margin classifier (Siena et al., 2013) and transductive learning (Zhu et al., 2014), are also explored. Further, deep learning–based approaches are also proposed for heterogeneous matching to learn shared representation (Yi et al., 2015), to leverage large homogeneous data (Reale et al., 2016), to learn using limited data (Hu et al., 2018), to facilitate transfer learning (Liu et al., 2016), performing face hallucination *via* disentangling (Duan et al., 2020), and learning deep models using Wasserstein distance (He et al., 2019). Deng Z. et al. (2019) extend MCA to utilize convolutional neural networks for heterogeneous matching. Most recent representation learning methods have a large parameter space, hence require enormous amounts of data for training models for heterogeneous matching. Nevertheless, learned face representations from such approaches are found to be very effective (Taigman et al., 2014; Majumdar et al., 2016; Wu et al., 2018; Deng J. et al., 2019).

In the literature, we identify a scope for improving statistical techniques for heterogeneous matching scenarios. Specifically, we observe that for heterogeneous matching task, modeling of intra-view variability is not critical, as the task always involves matching an inter-view/heterogeneous face pair. The objective functions of the proposed approaches differ from the literature in focusing only on the inter-view variability. To this end, we present two subspace-based classifiers aiming at reducing the inter-view intra-class variability and increasing the inter-view inter-class variability for heterogeneous face recognition. Specifically, in this article, we

• propose heterogeneous discriminant analysis (HDA) and its nonlinear kernel extension (KHDA),
• demonstrate the effectiveness of these HDA and KHDA using multiple features on three challenging heterogeneous face recognition scenarios: matching visible to near-infrared images, matching cross-resolution face images, and matching digital photo to composite sketch, and
• utilize deep learning–based features and show that combined with the proposed HDA and KHDA, they yield impressive heterogeneous matching performance.

## Heterogeneous Discriminant Analysis

To address the issue of heterogeneity in face recognition, we propose a discriminant analysis–based approach. In this context, the heterogeneity can arise due to factors such as spectrum variations as shown in Figure 1. The same individual may appear somewhat different in two different spectrums. While a feature extractor may filter out some of the heterogeneity, most feature extractors are not designed to be heterogeneity invariant. Therefore, for practical purposes, the heterogeneity of the source image may be retained in the extracted features.

By definition, the end goal of heterogeneous matching is always a cross-view comparison, for example, VIS to NIR matching and never intra-view comparison, for example, VIS to VIS matching. Therefore, the cross-view information would contain stronger cues for the task than the intra-view information. In other words, optmizing the intra-view variation may have limited utility. It is our hypothesis that incorporating only the cross-view (e.g., cross-spectral) information along with intra- and inter-class variability can improve heterogeneous matching. The proposed heterogeneous discriminant analysis is inspired from the formulation of linear discriminant analysis. Therefore, we first briefly summarize the formulation and limitations of linear discriminant analysis (LDA) followed by presenting the details of HDA.

Traditionally, intra- and inter-class variabilities are represented using within- $S_{W}=\sum_{i=1}^{c}\sum_{j=1}^{n_{i}}\left(x_{i,j}-\mu_{i}\right){\left(x_{i,j}-\mu_{i}\right)}^{T}$ and between-class scatter matrices $S_{B}=\sum_{i=1}^{c}\sum_{l=i+1}^{c}\left(\mu_{i}-\mu_{l}\right){\left(\mu_{i}-\mu_{l}\right)}^{T}$; where *c* is the total number of classes, $n_{i}$ is the number of samples in $i^{th}$ class, $x_{i,j}$ is the $j^{th}$ sample of the $i^{th}$ class, and $\mu_{i}$ is the mean of the $i^{th}$ class. The Fisher criterion $J\left(w\right)=\left|w^{T}S_{B}w\right|/\left|w^{T}S_{W}w\right|$ attempts to find the projection directions that minimize the intra-class variability and maximize the inter-class variability in the projected space.

**FIGURE 2:**
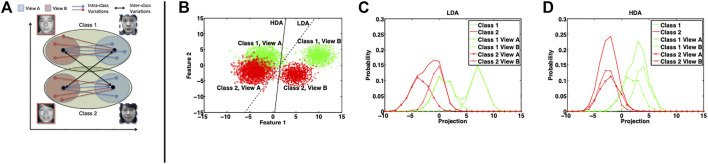
**(A)** Graphical interpretation of HDA and **(B–D)** illustration of the effectiveness of HDA with multiple views. Class 1 and 2 are generated using Gaussian mixture of two modes resulting in two views. **(B)** represents the scatter plot and the projection directions obtained using LDA and HDA (without regularization). The histograms of projections of data samples on the LDA and HDA directions are shown in **(C)** and **(D)**, respectively.

The way the scatter matrices are defined ensures that all the samples are as close to the corresponding class mean as possible and that class means are as apart as possible. Any new sample resembling the samples of a certain class would get projected near the corresponding class mean. LDA attempts to optimize the projection directions assuming that the data conforms to a normal distribution. Obtaining such a projection space is useful when the samples to be compared are homogeneous, that is, there is no inherent difference in the sample representation. Even if we assume that each view of each class is normally distributed in itself, the restrictive constraint of LDA is not satisfied. As shown in Figure 2, when provided with a multi-view or heterogeneous data, the projection directions obtained from LDA may be suboptimal and can affect the classification performance. Therefore, for heterogeneous matching problems, we propose to incorporate the view information while computing the between- and within-class scatter matrices.

The formulation of the proposed heterogeneous discriminant analysis is described in the following two stages: 1. adaptation of scatter matrices and 2. analytical solution.

### Adaptation of Scatter Matrices

Let $x_{i,j}^{a}$ and $x_{i,j}^{b}$ denote the two views (A and B) of the $j^{\text{th}}$ sample of the $i^{\text{th}}$ class, respectively, and $n_{i}^{a}$ and $n_{i}^{b}$ represent the number of samples in view A and B of the $i^{\text{th}}$ class, respectively. $\chi_{i}^{a}=\left\{x_{i,j}^{a}|1\leq j\leq n_{i}^{a}\right\}$ represents the samples in view A of $i^{\text{th}}$ class. For example, $\chi_{i}^{a}$ represents the visible spectrum face images of $i^{\text{th}}$ subject, and $\chi_{i}^{b}$ represents the near-infrared spectrum face images of the subject.

_•_ $\chi_{1}^{a}\;-\;\chi_{1}^{a}\;\text{and}\;\chi_{1}^{a}\;-\;\chi_{1}^{b}$ are examples of match pairs, that is, face images in a pair belong to the same subject.
_•_ $\chi_{1}^{a}\;-\;\chi_{2}^{b}\;\text{and}\;\chi_{1}^{a}\;-\;\chi_{2}^{b}$ are examples of non-match pairs consisting of face images of different subjects.
_•_ $\chi_{1}^{a}\;-\;\chi_{1}^{a}\;\text{and}\;\chi_{1}^{b}\;-\;\chi_{2}^{b}$ represent intra-view pairs where face images belong to the same view.
_•_ $\chi_{1}^{a}\;-\;\chi_{1}^{b}\;\text{and}\;\chi_{1}^{b}\;-\;\chi_{2}^{a}$ are examples of inter-view pairs, that is, face images in a pair belong to different view.

There can be four kinds of information: i) inter-class intra-view difference, ii) inter-class inter-view difference, iii) intra-class intra-view difference, and iv) intra-class inter-view difference. Optimizing the intra-view (homogeneous) distances would not contribute in achieving the goal of efficient heterogeneous matching. Therefore, the scatter matrices should be defined such that the objective function reduces the heterogeneity (inter-view variation) along with improving the classification accuracy. The distance between the inter-view samples of the non-matching class should be increased and the distance between inter-view samples of the matching class should be decreased. With this hypothesis, we propose the following two modifications in the scatter matrices for heterogeneous matching:

Inter-class inter-view difference encodes the difference between different views of two individuals (e.g., $\chi_{1}^{a}\;-\;\chi_{2}^{b}\;\text{and}\;\chi_{1}^{b}\;-\;\chi_{2}^{a}$ pairs). This can be incorporated in the between-class scatter matrix.

Intra-class inter-view difference encodes the difference between two different views of one person (e.g., $\chi_{1}^{a}\;-\;\chi_{1}^{b}\;\text{and}\;-\chi_{2}^{b}-\;\chi_{2}^{a}$ pairs). This can be incorporated in the within-class scatter matrix. (see Figure 2)

Incorporating these yields a projection space in which same-class samples from different views are drawn closer, thereby fine tuning the objective function for heterogeneous matching. The heterogeneous between-class scatter matrix ($S_{HB}$) encodes the difference between different views of different classes

$$\begin{array}{l} S_{HB}=\overset{c}{\underset{i=1}{\sum }}\overset{c}{\underset{l=1,l\neq i}{\sum }}p_{i}^{a}p_{l}^{b}\left(\mu_{i}^{a}-\mu_{l}^{b}\right){\left(\mu_{i}^{a}-\mu_{l}^{b}\right)}^{T}\\ \mu_{i}^{k}=\frac{1}{n_{i}^{k}}\underset{j}{\sum }x_{i,j}^{k},\;p_{i}^{k}=\frac{n_{i}^{k}}{n^{a}+n^{b}},\;k\in \left\{a,b\right\} \end{array}$$ (1)

Here, $\mu_{i}^{a}$ and $p_{i}^{a}$ are the mean and prior of view A of class *i*, respectively; $n^{a}$ represents the number of samples in view A. Similarly, $\mu_{i}^{b}$ and $p_{i}^{b}$ represent the mean and prior of view B of class *i*, respectively; $n^{b}$ represents the number of samples in view B. $n_{i}^{a}$ and $n_{i}^{b}$ represent the number of samples in view A and B of the $i^{th}$ class, respectively, and *c* represents the total number of classes. Note that, unlike CCA, the number of samples does not have to be equal in both views. The within-class scatter matrix $S_{HW}$ is proposed as

$$S_{HW}=\overset{c}{\underset{i=1}{\sum }}\left(\frac{1}{n_{i}^{a}}\overset{n_{i}^{a}}{\underset{j=1}{\sum }}\left(x_{i,j}^{a}-\mu_{i}^{b}\right){\left(x_{i,j}^{a}-\mu_{i}^{b}\right)}^{T}+\frac{1}{n_{i}^{b}}\overset{n_{i}^{b}}{\underset{j=1}{\sum }}\left(x_{i,j}^{b}-\mu_{i}^{a}\right){\left(x_{i,j}^{b}-\mu_{i}^{a}\right)}^{T}\right)$$ (2)

Since the proposed technique encodes data heterogeneity in the objective function and utilizes the definitions of between- and within-class scatter matrices, it is termed as heterogeneous discriminant analysis. Following the Fisher criterion, the objective function of HDA is proposed as

$$w=\arg\underset{w}{\max}\;J\left(w\right)=\arg\underset{w}{\max}\frac{\left|w^{T}S_{HB}w\right|}{\left|w^{T}S_{HW}w\right|}$$ (3)

The optimization problem in Eq. 3 is modeled as a generalized eigenvalue decomposition problem which results into a closed-form solution such that *w* is the set of top eigenvectors of $S_{HW}^{-1}S_{HB}$. The geometric interpretation of HDA in Figure 2 shows that the objective function in Eq. 3 tries to achieve the following in the projected space: 1) Bring samples $\chi_{1}^{a}$ closer to mean $\mu_{1}^{b}$ of $\chi_{1}^{b}$ and vice versa; and similarly for class 2. This reduces the inter-view distance within each class, for example, the projections of visible and NIR images of the same person become similar. 2) Increase the distance between mean $\mu_{1}^{a}$ of $\chi_{1}^{a}$ and mean $\mu_{2}^{b}$ of $\chi_{2}^{b}$; and similarly increase the distance between mean of $\chi_{1}^{b}$ and mean of $\chi_{2}^{a}$, that is, the projections of mean visible face image of a subject become different from the mean NIR face image of another subject. The proposed way of encoding inter- (Eq. 1) and intra-class (Eq. 2) variations in the heterogeneous scenario requires that both the views are of the same dimensionality. In the application domain of face recognition, this is usually not an unrealistic constraint as, in practice, same kind of features, with same dimensionality, are extracted from both the views (Dhamecha et al., 2014).

In some applications including face recognition, the number of training samples is often limited. If the number of training samples is less than the feature dimensionality, it leads to problems such as singular within-class scatter matrix. In the literature, it is also known as the small sample size problem and shrinkage regularization is generally used to address the issue (Friedman, 1989). Utilizing the shrinkage regularization, Eq. 3 is updated as

$$J\left(w\right)=\frac{\left|w^{T}S_{HB}w\right|}{\left|w^{T}\left(\left(1-\lambda \right)S_{HW}+\lambda I\right)w\right|}$$ (4)

Here, *I* represents the identity matrix and λ is the regularization parameter. Note that λ=0 results in no regularization, whereas λ=1 results into not utilizing the within-class scatter matrix $S_{HW}$.

To visualize the functioning of the proposed HDA as opposed to LDA, the distributions of the projections obtained using LDA and HDA are shown in Figure 2. Table 1 presents a quantitative analysis in terms of the overlap between projections of views of both classes. The overlap between two histograms is calculated as $\sum_{m}\text{min}\left(h_{1}\left(m\right),h_{2}\left(m\right)\right)$, where $h_{1}\left(m\right)$ and $h_{2}\left(m\right)$ are the values of the $m^{th}$ bin of the first and second histograms, respectively. In the ideal case, the projections of different views of the same class should completely overlap (i.e., area of overlap 0.5) and the projections of the views of different classes should be nonoverlapping (i.e., area of overlap 0). Since LDA does not take into account the view information, the overlap between projections of both classes is large. Further, it is interesting to note that LDA yields a significant overlap of 0.351 between view A of class 1 and view B of class 2. Such overlap can deteriorate the heterogeneous matching performance. In the heterogeneous analysis (last two rows of Table 1), the overlap between projections of two views of the same class is relatively low. Note that view A and view B of class 1 result in two individual peaks. This also increases the intra-class variation, that is, projection distributions of both classes are spread rather than peaked. HDA yields better projection directions with less than 50% of inter-class overlap compared to LDA. For the homogeneous matching scenarios (fourth and fifth rows), HDA has marginally poor overlap compared to LDA. However, for the heterogeneous scenarios, the overlap of HDA is significantly lower for non-match pair of view A class 1–view B class 2 (seventh row) and higher for match pairs (last two rows). For the view A class 2–view B class 1 (eighth row), the numbers are slightly poorer for HDA; however, the difference is small enough to be neglected in context of the overlap metrics of other three pairs.

**TABLE 1:** Analyzing the overlap of projection distributions in Figures 2. LDA vs HDA comparison indicates that ignoring intra-view differences could be beneficial for heterogeneous matching.

Pair	Overlap		
	Ideal	LDA	HDA
**Overall**			
Class 1–class 2	0.000	0.356	0.159
**Homogeneous**			
View A class 1–view A class 2	0.000	0.110	0.135
View B class 1–view B class 2	0.000	0.005	0.013
**Heterogeneous**			
View A class 1–view B class 2	0.000	0.351	0.076
View A class 2–view B class 1	0.000	0.000	0.034
View A class 1–view B class 1	0.500	0.025	0.261
View A class 2–view B class 2	0.500	0.174	0.429

The time complexity of computing $S_{HB}$ and $S_{HW}$ is $O\left(nd^{2}\right)$ and $O\left(c^{2}d^{2}\right)$, respectively. The generalized eigenvalue decomposition in Eq. 3 has time complexity of $O\left(d^{3}\right)$, where *n*, *d*, and *c* are the number of training samples, feature dimensionality, and number of classes, respectively.

### Nonlinear Kernel Extension

We further analyze the objective function in Eq. 3 to adapt it for nonlinear transformation x→ϕ(x). Using the representer theorem (Schölkopf et al., 2001), the projection direction in *w* can be written as linear sum of the transformed samples, that is, $w=\sum_{p=1}^{n^{a}}\alpha_{p}\phi \left(x_{p}^{a}\right)+\sum_{q=1}^{n^{b}}\beta_{q}\phi \left(x_{q}^{b}\right)$. Using this property, the Eq. 4 can be rewritten as^2^

$$J\left(\alpha ,\beta \right)=\left(\frac{\left|\left[\alpha^{T}\beta^{T}\right]M_{\text{*}}\left[\begin{array}{c} \alpha \\ \beta \end{array}\right]\right|}{\left|\left[\alpha^{T}\beta^{T}\right]\left[\left(1-\lambda \right)N_{\text{*}}+\lambda \right]\left[\begin{array}{c} \alpha \\ \beta \end{array}\right]\right|}\right)$$ (5)

where $M_{\text{*}}$ and $N_{\text{*}}$ are analogous to $S_{HB}$ and $S_{HW}$, respectively, and are defined as

$$M_{*}=\overset{c}{\underset{i=1}{\sum }}\overset{c}{\underset{l=1,l\neq i}{\sum }}p_{i}^{a}p_{l}^{b}\left[\begin{array}{c} ℳA_{i}^{a}-ℳA_{l}^{b}\\ ℳ{ℬ}_{i}^{a}-ℳ{ℬ}_{l}^{b} \end{array}\right]{\left[\begin{array}{c} ℳA_{i}^{a}-ℳA_{l}^{b}\\ ℳ{ℬ}_{i}^{a}-ℳ{ℬ}_{l}^{b} \end{array}\right]}^{T}$$

$$N_{*}=\overset{c}{\underset{i=1}{\sum }}\left(\frac{1}{n_{i}^{a}}\overset{n_{i}^{a}}{\underset{j=1}{\sum }}\left[\begin{array}{c} MA_{i,j}^{a}-ℳA_{i}^{b}\\ M{ℬ}_{i,j}^{a}-ℳ{ℬ}_{i}^{b} \end{array}\right]{\left[\begin{array}{c} MA_{i,j}^{a}-ℳA_{i}^{b}\\ M{ℬ}_{i,j}^{a}-ℳ{ℬ}_{i}^{b} \end{array}\right]}^{T}+\frac{1}{n_{i}^{b}}\overset{n_{i}^{b}}{\underset{j=1}{\sum }}\left[\begin{array}{c} MA_{i,j}^{b}-ℳA_{i}^{a}\\ M{ℬ}_{i,j}^{b}-ℳ{ℬ}_{i}^{a} \end{array}\right]{\left[\begin{array}{c} MA_{i,j}^{b}-ℳA_{i}^{a}\\ M{ℬ}_{i,j}^{b}-ℳ{ℬ}_{i}^{a} \end{array}\right]}^{T}\right)$$

where ${\left(ℳ{ℬ}_{i}^{a}\right)}_{q}=\frac{1}{n_{i}^{a}}\overset{n_{i}^{a}}{\underset{s=1}{\sum }}K\left(x_{q}^{b},x_{i,s}^{a}\right)$ and ${\left(M{ℬ}_{i,j}^{a}\right)}_{q}=K\left(x_{q}^{b},x_{i,j}^{a}\right)$, where *K* is a kernel function. In this work, we use the Gaussian kernel function. Eq. 5 with linear kernel is equivalent to Eq. 4. However, if d<n, the criterion in Eq. 4 is computationally more efficient than Eq. 5 but if d>n, Eq. 5 is computationally more efficient than Eq. 4.

## Proposed Cross-View Face Recognition Approach

**FIGURE 3:**
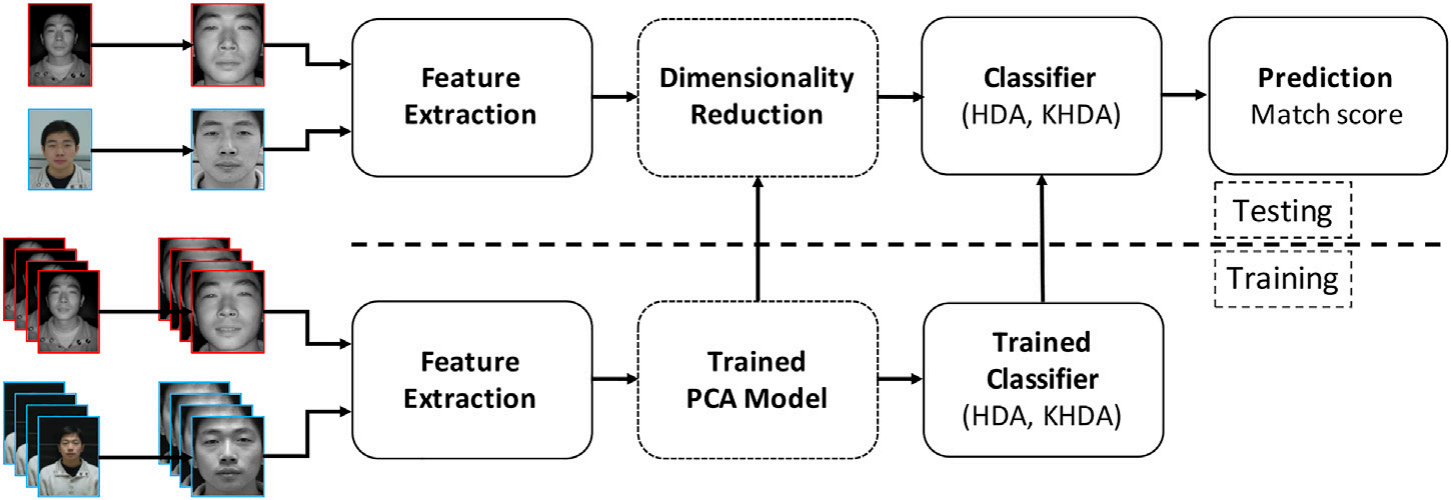
Illustrating the steps involved in the face recognition pipeline with the proposed HDA and KHDA.

The main objective of this research is to utilize the proposed heterogeneity-aware classifiers in conjunction with robust and unique features for heterogeneous face recognition. Figure 3 showscases the steps involved in the face recognition pipeline. From the given input image, the face region is detected using a Haar face detector or manually annotated (for digital sketches) eye coordinates. It is our assertion that the proposed HDA and KHDA should yield good results with both handcrafted and learnt representations. Based on our formulation, to a large extent, HDA and KHDA should help obtain heterogeneity invariant representation of features. Therefore, the lesser heterogeneity invariant a feature is, the greater should be the extent of improvement by HDA and KHDA. Arguably, the learned features are more sophisticated and heterogeneity invariant compared to handcrafted features. Therefore, in this research, we have performed experiments with features of both types for detailed evaluation. In the literature, it has been observed that histogram of oriented gradients (HOG) and local binary patterns (LBP) are commonly used handcrafted features for heterogeneous face matching (Klare and Jain, 2013, 2010). Dhamecha et al. (2014) compared the performance of different variants of HOG and showed that DSIFT (Lowe, 2004) yields the best results. Therefore, among handcrafted features, we have demonstrated the results with DSIFT (extracted at keypoints on uniform grid and landmark points). For learnt representation, we use local class sparsity–based supervised encoder (LCSSE) (Majumdar et al., 2016), LightCNN (Wu et al., 2018), and ArcFace (Deng J. et al., 2019). For LightCNN (LightCNN29V2) and ArcFace, both the models pretrained on MS-Celeb 1M dataset are utilized as feature extractor. In this research, we have used the pretrained LCSSE model and fine-tuned with the training samples for each case study.

As shown in Figure 3, once the features are obtained, they are projected on to a PCA space (preserving 99% eigenenergy), followed by projecting onto the c−1 dimensional HDA or KHDA space. It is to be noted that learning of PCA subspace does not use class labels, whereas HDA and KHDA training utilize identity labels and the view labels. Finally, distance score between gallery and probe feature vectors is computed using cosine distance measure.

## Experimental Evaluation

The effectiveness of the proposed heterogeneous discriminant algorithm is evaluated for three different case studies of heterogeneous face recognition: 1) visible to near-infrared matching, 2) cross-resolution face matching, and 3) composite sketch (CS) to digital photo (DP) matching. For all three case studies, we have used publicly available benchmark databases: CASIA NIR-VIS 2.0 (Li et al., 2013), CMU Multi-PIE (Gross et al., 2010), and e-PRIP composite sketch (Han et al., 2013; Mittal et al., 2014). Table 2 summarizes the characteristics of the three databases. The experiments are performed with existing and published protocols so that the results can be directly compared with reported results.

**TABLE 2:** Datasets utilized for evaluating the proposed HDA and KHDA on three heterogeneous face recognition challenges.

Case Study	Gallery	Probe	Dataset	#Images	#Subjects	
						Total Training: Testing (Protocol)
Cross-spectral	VIS	NIR	CASIA NIR-VIS-2.0 (Li et al., 2013)	17,850	725	357 : 358 (Li et al., 2013)
Cross-resolution	HR	LR	CMU Multi-PIE (Gross et al., 2010)	18,420	337	100 : 227 (Bhatt et al., 2012; Bhatt et al., 2014)
Photo to sketch	DP	CS	e-PRIP composite sketch (Han et al., 2013; Mittal et al., 2014)	246	123	48 : 75 (Mittal et al., 2014)

### Cross-Spectral (Visible–NIR) Face Matching

Researchers have proposed several algorithms for VIS to NIR matching and primarily used the CASIA NIR-VIS 2.0 face dataset (Li et al., 2013). The protocol defined for performance evaluation consists of 10 splits of train and test sets for random subsampling cross-validation. As required by the predefined protocol, results are reported for both identification (mean and standard deviation of rank-1 identification accuracy) and verification (GAR at 0.1% FAR).

The images are first detected and preprocessed. Seven landmarks (two eye corners, three points on nose, and two lip corners) are detected (Everingham et al., 2009) from the input face image and geometric normalization is applied to register the cropped face images. The output of preprocessing is grayscale face images of size 130×150 pixels. All the features^3^ are extracted from geometrically normalized face images. We evaluate the effectiveness of HDA over LDA. To compare the results with LDA, the pipeline shown in Figure 3 is followed with the exception of using LDA instead of HDA. The results are reported in Table 3 and the key observations are discussed below.^4^

**TABLE 3:** Rank-1 identification accuracy for visible to near-infrared face matching on the CASIA NIR–VIS 2.0 database (Li et al., 2013).

Algorithm		DSIFT	LCSSE	LightCNN	ArcFace
W/O DA	Eucl	12.6±0.9	50.3±8.3	95.7±0.3	97.1±0.4
	Cos	19.6±1.4	51.6±7.8	96.9±0.3	97.4±0.5
LDA	Eucl	56.7±2.2	82.3±4.8	96.8±0.3	98.2±0.9
	Cos	80.4±1.7	88.9±3.2	98.1±0.5	98.5±0.6
HDA	Eucl	58.0±2.1	95.2±1.7	96.3±0.5	99.1±0.2
	Cos	81.0±1.9	96.8±0.9	98.1±0.3	99.3±0.2

#### Discriminative Learning using HDA

As shown in Table 3, without discriminant analysis (LDA or HDA), the performance of individual features is lower. The deep learning–based LCSSE yields around 50% rank-1 accuracy. The LightCNN and ArcFace features yield impressive rank-1 accuracy of about 95% and 97%, respectively, which shows their superior feature representation. The next experiment illustrates the effect of applying LDA on individual features. Table 3 shows that LDA improves the accuracy up to 60%. Comparing the performance of HDA with LDA shows that HDA outperforms LDA. Utilizing HDA in place of LDA for discriminative learning improves the results up to 12.9%. The HDA and LDA performance is very high and almost same for LightCNN, which may point toward its spectrum-invariant representation capabilities. For ArcFace, although small, a consistently progressive improvement of about 1% is observed between raw features, LDA, and HDA, respectively. Understandably, if the feature is spectrum-invariant, the benefits of heterogeneity-aware classifier are expected to be limited. The improvement provided by HDA can be attributed to the fact that it learns a discriminative subspace specifically for heterogeneous matching. Similar to the toy example shown in Figure 2, it can be asserted that the multi-view information yields different clusters in the feature space. Under such scenarios, since the fundamental assumption of Gaussian data distribution is not satisfied, LDA can exhibit suboptimal results. However, by encoding the view label information, HDA is able to find better projection space, thereby yielding better results.

#### Effect of HDA across Features

The results show that the proposed HDA improves the accuracy of DSIFT and LCSSE features by 40–60%. For instance, applying LCSSE with HDA improves the results by around 45%. As discussed earlier, even the raw LightCNN and ArcFace features yield very high performance, leaving very little room of improvement by LDA or HDA projections.

#### Direction vs Magnitude in Projection Space

Cosine distance encodes only the difference in direction between samples, whereas the Euclidean distance encodes both direction and magnitude. For the given experiment, as shown in Table 3, cosine distance generally yields higher accuracy over Euclidean distance. This shows that for heterogeneous matching, the magnitude of projections may not provide useful information and only directional information can be used for matching.

#### Optimum Combination

From the above analysis, it can be seen that the proposed HDA in combination with DSIFT features and cosine distance measure yields an impressive 81% for a handcrafted feature. ArcFace features with HDA and cosine distance measure yield the best results. However, LightCNN and LCSSE are also within 3% of it. For the remaining experiments (and other case studies), we have demonstrated the results with DSIFT, LCSSE, LightCNN, and ArcFace features and cosine distance measure along with proposed heterogeneity-aware classifiers.

#### Comparison with Existing Algorithms

We next compare the results of the proposed approaches with the results reported in the literature. Comparative analysis is shown with a leading commercial off-the-shelf (COTS) face recognition system, FaceVACS^5^, and 20 recently published results. Table 4 shows that with pixel values as input, the proposed HDA approach outperforms other existing algorithms. For example, MvDA with pixel values yields 41.6% rank-1 identification accuracy and 19.2% GAR at 0.1% FAR, whereas the proposed approach yields similar rank-1 accuracy with lower standard deviation and much higher GAR of 31.4%. Further, Table 4 clearly^6^ demonstrates the performance improvement due to the proposed HDA and its nonlinear kernel variant KHDA. KHDA with learnt representation LCSSE and HDA with LightCNN yield almost equal identification accuracy. However, our best results are obtained with ArcFace with KHDA at 99.4% rank-1 and 99.1% GAR@FAR=0.1%. The reported results are comparable to the recently published state of the art.

**TABLE 4:** Comparing the face recognition performance of the proposed and some existing algorithms for VIS to NIR face matching on CASIA NIR–VIS 2.0 dataset.

Algorithm	Year	Rank-1	GAR
		Accuracy (%)	@ FAR = 0.1%
FaceVACS (Dhamecha et al., 2014)	2014	58.6±1.2	52.9
**Pixels as Features**			
CCA^a^ (Hardoon et al., 2004)	2004	28.5±3.4	10.8
PLS^a^ (Sharma and Jacobs, 2011)	2011	17.7±1.9	2.3
CDFE^a^ (Lin and Tang, 2006)	2006	27.9±2.9	6.9
MvDA^a^ (Kan et al., 2016)	2012	41.6±4.1	19.2
GMLDA^a^ (Sharma et al., 2012)	2012	23.7±1.4	5.1
GMMFA^a^ (Sharma et al., 2012)	2012	24.8±1.1	7.6
PCA+Symmetry+HCA (Li et al., 2013)	2013	23.7±1.9	19.3
PIXEL+HDA	-	41.4±1.3	31.4
**Other Features/Approaches**			
DSIFT+SDA (H=2) (Zhu and Martinez, 2006)	2006	75.7±1.2	54.8
Gabor+RBM+Remove 11 PC (Yi et al., 2015)	2015	86.2±1.0	81.3
C-DFD (s=3)^a^ (Lei et al., 2014)	2014	65.8±1.6	46.2
CDFL (s=3) (Jin et al., 2015)	2015	71.5±1.4	55.1
C-CBFD+LDA (Lu et al., 2015)	2015	81.8±2.3	47.3
Joint Dictionary Learning (Juefei-Xu et al., 2015)	2015	78.5±1.7	85.8
Saxena and Verbeek (2016)	2016	85.9±0.9	78.0
Reale et al. (2016)	2016	87.1±0.9	74.5
TRIVET (Liu et al., 2016)	2016	95.7±0.5	91.0
MTC-ELM (Jin et al., 2016)	2016	89.1	-
Lezama et al. (2017)	2017	89.6±0.9	-
He et al. (2017)	2017	95.8±0.8	94.0
Gabor+HJB (Shi et al., 2017)	2017	91.7±0.9	89.9
G-HFR (Peng et al., 2017)	2017	85.3±0.0	-
Frankenstein (Hu et al., 2018)	2018	85.1±0.8	-
LightCNN (Wu et al., 2018)	2018	96.7±0.2	94.8
WCNN (He et al., 2019)	2019	98.7	98.4
MC-CNN (Deng et al., 2019b)	2019	99.2±0.2	-
RGM+NAU+C-softmax (Cho et al., 2020)	2020	99.3±0.1	98.9
PACH (Duan et al., 2020)	2020	98.9±0.2	98.3
DSIFT+HDA	-	81.0±1.9	62.8
DSIFT+KHDA	-	83.1±1.7	62.1
LCSSE+HDA	-	96.8±0.9	93.1
LCSSE+KHDA	-	98.1±0.5	94.3
LightCNN+HDA	-	98.1±0.3	96.5
ArcFace+HDA	-	99.3±0.2	98.8
ArcFace+KHDA	-	99.4±0.1	99.1

a represents the results reported in Jin et al. (2015), Lu et al. (2015). Other cited results as reported in their corresponding publications.

Also, LCSSE+KHDA and LightCNN+HDA achieve 94.3% and 96.5% GAR at 0.1% FAR, respectively. Also note that, in a fair comparison, DSIFT features with the proposed KHDA also yield results comparable to other non-deep learning–based approaches.

### Cross-Resolution Face Matching

Cross-resolution face recognition entails matching high-resolution gallery images with low-resolution probe images. In this scenario, high resolution and low resolution are considered as two different views of a face image. We compare our approach with Bhatt et al. (2012, 2014) as they have reported one of the best results for the problem. We follow their protocol on CMU Multi-PIE database (Gross et al., 2010). Each image is resized to six different resolutions: 16×16, 24×24, 32×32, 48×48, 72×72, and 216×216. In total, $\left(\begin{array}{c} 6\\ 2 \end{array}\right)=15$ cross-resolution matching scenarios are considered. For every person, two images are selected and images pertaining to 100 subjects are utilized for training, whereas the remaining 237 subjects are utilized for testing. The results are reported in Table 5. Results for ArcFace+KHDA are similar to ArcFace+HDA, hence not reported here. Since the protocol (Bhatt et al., 2012, 2014) does not involve cross-validation, error intervals are not reported.

**TABLE 5:** Rank-1 identification accuracy of the proposed HDA, KHDA and existing algorithms, Cotransfer Learning (CTL) and a commercial off-the-shelf (COTS) (Bhatt et al., 2012, 2014), DSIFT (Lowe, 2004), LCSSE (Majumdar et al., 2016), LightCNN, and ArcFace on CMU Multi-PIE database (Gross et al., 2010) with different gallery and probe image sizes.

Probe res.	CTL	COTS	DSIFT		LCSSE		LightCNN	ArcFace
			HDA	KHDA	HDA	KHDA	HDA	HDA
**Gallery: 216 × 216**								
72×72	81.0	99.5	94.1	95.4	95.8	97.0	100	100
48×48	79.7	98.1	92.4	94.1	93.7	95.3	100	100
32×32	65.3	97.4	89.0	90.7	92.0	93.2	99.6	100
24×24	37.7	54.5	87.3	85.7	89.0	89.5	92.0	95.0
16×16	23.6	10.9	37.6	37.6	61.2	62.5	35.0	46.0
**Gallery: 72 × 72**								
48×48	92.3	92.7	95.4	96.2	96.6	97.0	100	100
32×32	84.1	84.3	92.4	96.2	92.8	96.6	100	100
24×24	77.4	78.5	89.0	91.6	93.2	94.1	95.4	98.2
16×16	72.4	72.8	44.3	54.9	73.4	75.1	39.2	52.4
**Gallery: 48 × 48**								
32×32	61.8	96.8	95.4	97.1	96.2	97.9	100	100
24×24	57.1	75.9	95.4	94.9	96.6	97.5	89.9	94.8
16×16	32.9	6.4	73.8	71.3	77.2	78.1	34.6	50.0
**Gallery: 32 × 32**								
24×24	45.7	78.4	94.9	94.5	95.8	96.2	98.7	100
16×16	28.1	5.4	88.6	86.1	90.3	91.1	50.6	62.4
**Gallery: 24 × 24**								
16×16	43.2	16.3	85.7	85.2	87.3	89.0	56.5	68.8

It can be seen that LCSSE+KHDA outperforms the cotransfer learning (Bhatt et al., 2012, 2014) in all the cross-resolution matching scenarios. For example, when 48×48 pixel gallery images are matched with probe images of 32×32, 24×24, and 16×16 pixels, performance improvement of about 30%–40% is observed. LightCNN and ArcFace yield even higher identification accuracy, except when the probe image is 16×16. We believe that the feature extractor is unable to extract representative information at these resolutions. Analyzing the results across resolutions shows that the accuracy reduces with increase in resolution difference between the gallery and probe images. FaceVACS yields impressive performance when the size of both gallery and probe are higher than 32×32. However, the performance deteriorates significantly with decrease in the gallery image size and with increase in the resolution difference. Generally, the performance of the proposed HDA and/or KHDA is less affected due to resolution difference in comparison to FaceVACS and CTL. We have also observed that for cross-resolution face recognition, learned features (LCSSE, LightCNN, and ArcFace) show higher accuracies compared to DSIFT with a difference of up to 25%.

### Digital Photo to Composite Sketch Face matching

In many law enforcement and forensic applications, software tools are used to generate composite sketches based on eyewitness description and the composite sketch is matched against a gallery of digital photographs. Han et al. (2013) presented a component-based approach followed by score fusion for composite to photo matching. Later, Mittal et al. (2014, 2013, 2015, 2017) and Chugh et al. (2013) presented learning-based algorithms for the same. Klum et al. (2014) presented FaceSketchID for matching composite sketches to photos.

**FIGURE 4:**
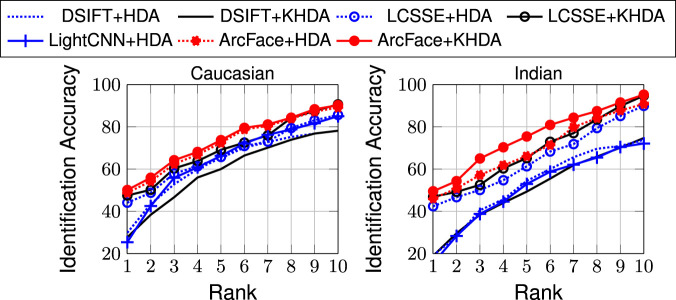
CMC curves for composite sketch to digital photo matching on the e-PRIP composite sketch dataset (Han et al., 2013; Mittal et al., 2014).

For this set of experiments, we utilize the e-PRIP composite sketch dataset (Han et al., 2013; Mittal et al., 2014). The dataset contains composite sketches of 123 face images from the AR face dataset (Martinez, 1998). It contains the composite sketches created using two tools, Faces and IdentiKit^7^. The PRIP dataset (Han et al., 2013) originally has composite sketches prepared by a Caucasian user (with IdentiKit and Faces softwares) and an Asian user (with Faces software). Later, the dataset is extended by Mittal et al. (2014) by adding composite sketches prepared by an Indian user (with Faces software) which is termed as the e-PRIP composite sketch dataset. In this work, we use composite sketches prepared using Faces software by the Caucasian and Indian users as they are shown to yield better results compared to other sets (Mittal et al., 2014, 2013). The experiments are performed with the same protocol as presented by Mittal et al. (2014). Mean identification accuracies, across five random cross-validations, at rank-10 are reported in Table 6, and Figure 4 shows the corresponding CMC curves.

**TABLE 6:** Results for composite sketch to photo matching.

Algorithm	Rank-10 Accuracy (%)	
	Faces (Caucasian)	Faces (Indian)
Mittal et al. (2015)	56.0±2.1	60.2±2.9
Mittal et al. (2017)	59.3±0.8	58.4±1.1
COTS (Mittal et al., 2014)	11.3±2.1	9.1±1.9
Saxena and Verbeek (2016)	-	65.6±3.7
DSIFT only	67.5±5.8	51.7±4.0
DSIFT+HDA	79.5±2.8	73.9±5.8
DSIFT+KHDA	78.6±3.4	74.6±3.8
LCSSE only	68.0±2.6	65.3±4.1
LCSSE+HDA	85.6±1.3	89.0±1.5
LCSSE+KHDA	89.6±1.9	94.7±1.0
LightCNN only	84.6±0.9	75.4±1.0
LightCNN+HDA	85.0±0.6	72.1±0.9
ArcFace only	86.5±0.2	80.6±1.3
ArcFace+HDA	89.1±0.6	90.8±1.1
ArcFace+KHDA	90.2±0.4	95.2±0.7

With the above mentioned experimental protocol, one of the best results in the literature has been reported by Mittal et al. (2017) with rank-10 identification accuracies of 59.3% (Caucasian) and 58.4% (Indian). Saxena and Verbeek (2016) have shown results with Indian users only and have achieved 65.5% rank-10 accuracy. As shown in the results, the proposed approaches, HDA and KHDA, with both DSIFT and LCSSE improve the performance significantly. Compared to existing algorithms, DSIFT demonstrates an improvement in the range of 11–23%, while LCSSE+HDA and LCSSE+KHDA improve the rank-10 accuracy by ∼30% with respect to state of the art (Saxena and Verbeek, 2016). Interestingly, LightCNN yields poorer performance compared to LCSSE in this case study. ArcFace yields the highest identification accuracy. Similar to previous results, this experiment also shows that application of HDA/KHDA improves the results of DSIFT, LCSSE, and ArcFace. However, the degree of improvement varies between handcrafted and learned features.

## Conclusion

In this research, we have proposed a discriminant analysis approach for heterogeneous face recognition. We formulate heterogeneous discriminant analysis which encodes view labels and has the objective function optimized for heterogeneous matching. Based on the analytical solution, we propose its kernel extension, KHDA. The proposed techniques are heterogeneity aware. Potentially, they can be applied on top of any features to get heterogeneity invariant representation, to an extent. Experiments are performed on three heterogeneous face matching problems, namely, visible to NIR matching, cross-resolution matchings, and digital photo to sketch, with handcrafted DSIFT and deep learning–based LCSSE, LightCNN, and ArcFace features. The results show that incorporating the proposed discriminant analysis technique consistently improves the performance of both learnt and handcrafted features, without increasing much to the computational requirements. The improvement is more pronounced in handcrafted features and provides an efficient way to improve their performance.

## Data Availability

Publicly available datasets were analyzed in this study. This data can be found here: http://www.cbsr.ia.ac.cn/english/HFB_Agreement/NIR-VIS-2.0_agreements.pdf, https://www.cs.cmu.edu/afs/cs/project/PIE/MultiPie/Multi-Pie/Home.html, https://www.iab-rubric.org/resources/eprip.html.

## Footnotes

1 The terms view and domain/modality are used synonymously in the heterogeneous face recognition literature.

2 Detailed formulation is in the supplementary document.

3 Results of LBP, HOG variants, and pixel are in supplementary document.

4 There is slight difference between LightCNN + W/O DA of Table 3 and LightCNN in Table 4, as former is our implementation and later is as reported in (Wu et al., 2018).

5
http://www.cognitec.com/technology.html

6 ROC in the supplementary document.

7 Faces: www.iqbiometrix.com, IdentiKit: www.identikit.net
